# Modelling to identify direct risks for New Zealand agriculture due to climate change

**DOI:** 10.1080/03036758.2024.2393295

**Published:** 2024-09-24

**Authors:** Linda Lilburne, Anne-Gaelle Ausseil, Abha Sood, Jing Guo, Edmar Teixeira, Indrakumar Vetharaniam, Tony van der Weerden, Hugh Smith, Andrew Neverman, Rogerio Cichota, Craig Phillips, Patricia Johnson, Steve Thomas, Robyn Dynes

**Affiliations:** a Manaaki Whenua – Landcare Research, Lincoln, New Zealand; b Ministry for the Environment, Wellington, New Zealand; c NIWA, Wellington, New Zealand; d Plant & Food Research, Lincoln, New Zealand; e Plant & Food Research, Palmerston North, New Zealand; f AgResearch, Invermay, New Zealand; g Manaaki Whenua – Landcare Research, Palmerston North, New Zealand; h AgResearch, Lincoln, New Zealand

**Keywords:** Hazards, vulnerabilities, arable, horticulture, pastoral

## Abstract

Climate change will affect New Zealand’s diverse range of climatic systems in different ways. The impacts on agriculture are expected to vary with geographical location and the specific biophysical requirements of different crops and agricultural systems. To improve our understanding of these impacts, key biophysical vulnerabilities for the main farming systems in New Zealand were identified and modelled using the daily projected climate scenario data. Results show high spatial variability but a general pattern of suitability ranges for crops moving south, and animal health issues intensifying and also moving south. Sediment loads are projected to increase, particularly in soft-rock hill country areas in the North Island. The modelling approach offers opportunities for analysing the temporal significance of projected changes, such as the timing and duration of drought, the effect on timing of phenological stages, the timing of pasture growth and the effect on animal farm systems.

## Introduction

### Background

The New Zealand Climate Change Risk Assessment (CCRA) report identifies the impact of climate change on primary industries and thus the economy as a major risk with the third highest urgency score (Ministry for the Environment 2020). New Zealand’s primary sector is vulnerable to a range of weather-related risks, and this could be exacerbated by climate change, with the prospect of declining yields and profitability, and adverse socio-economic impacts as a consequence of unfavourable changes to temperature and rainfall patterns (Hopkins et al. 2015; Cradock-Henry et al. 2019; Ausseil et al. 2019b). However, climate change could also provide new opportunities to diversify agricultural activities as the climate warms. Although projected temperature warming for New Zealand is less than the global average, change is still expected to have significant impacts because of our mild climate (Manning et al. 2015; Lawrence et al. 2022). These changes could affect agricultural production systems directly through:

− temperature regulation of crop growth and development (Hatfield and Prueger 2015) as well as soil-based processes that support plant growth (Orwin et al. 2015),
− altering rainfall patterns (Snyder 2017), and
− more acutely by modulating the virulence of pests and disease (Jones 2016; Trębicki et al. 2017; Wakelin et al. 2018; Mansfield et al. 2021).

Climate change will affect New Zealand’s diverse range of climatic systems in different ways, with impacts on agriculture expected to vary with geographical location and the specific requirements of different crops and agricultural systems (Warrick et al. 2001; Clark et al. 2012). Under climate change some areas may become less suitable for certain crops or farm systems, but new opportunities may arise elsewhere where low temperatures currently limit crop growth. Given the large spatial variability and crop specificity of impacts, farm systems may need to adopt locally tailored adaptation strategies to minimise risks and become more resilient. Information on the projected effects of climate change is essential for timely adaptation, including the option of land-use change (Clark et al. 2012).

**Figure 1.**
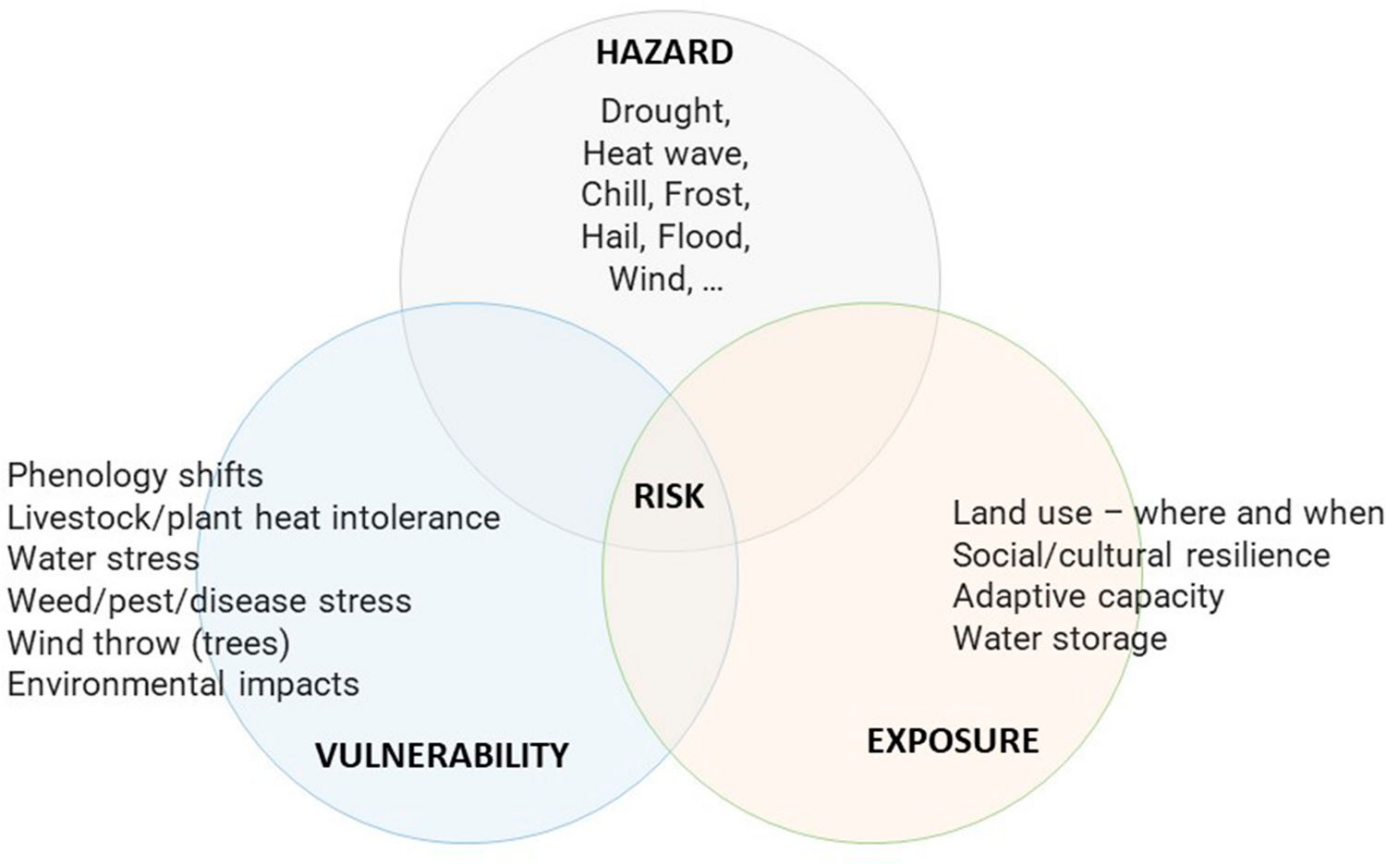
Risk framework (adapted from Openheimer et al. 2014).

We use the Intergovernmental Panel on Climate Change risk framework and definitions of hazard, vulnerability, exposure and risk (Openheimer et al. 2014) to help ensure a comprehensive assessment of the effects of climate change on agriculture (Figure 1). There are interactions between the various physical climate *hazards* (events and trends) and the *vulnerability* of the range of agricultural crops and farm systems in New Zealand. For example, the climate *hazard* of changing patterns in rainfall may be more or less risky depending on the *vulnerability* or sensitivity of a cropping system to the intensity or timing of drought or excess moisture. Similarly, changing rainfall patterns may alter *exposure* in locations where access to irrigation water is not reliable, thus increasing the risk of drought. Interactions between hazard, vulnerability, and exposure, the spatial and temporal variability of the climate response, and the uncertainty in the projections mean that modelling approaches are needed to gain an understanding of climate change impacts on agriculture in New Zealand.

### Risk framework

Because our focus is on understanding future land-use suitability, we have limited our work to the hazard and vulnerability components. We have not considered the exposure component because that would imply understanding actual current (and future) land uses and infrastructure, as well as the socio-economic-cultural context in New Zealand. We identified a set of hazards affecting vulnerabilities relevant to New Zealand agriculture, which functioned as an assessment of potential risks and opportunities (Table 1). These have been identified through consultation with experts and stakeholders (Ausseil et al. 2019a).

**Table 1.** Model outputs for some hazard and vulnerability risks to New Zealand agriculture (*: discussed in this paper; yellow cells: available from https://landuseopportunities.nz/; italics: other research outputs). ap = apple, av = avocado, bl = blueberry, ch = cherry, cn = chestnut, ki = kiwifruit, ma = maize, on = onions, pe = peas, po = potatoes, wh = wheat, wi = wine grape.

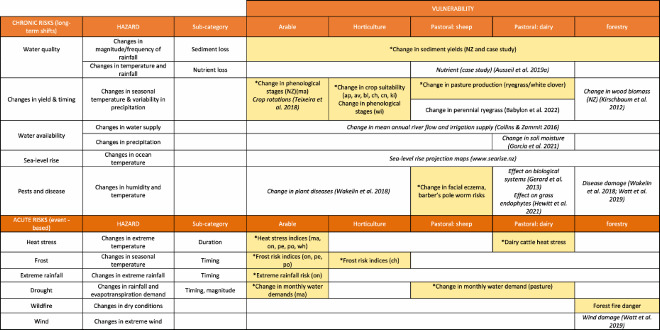

The risks focused on the physical impacts of climate change and were categorised according to the Task Force on Climate-related Financial Disclosures (TCFD^1^) into chronic risks (based on long-term shifts in climate patterns) and acute risks (event-driven risks such as extreme events). A biophysical modelling approach was then used to quantify these risks. Note that we focused on direct risks rather than indirect risks such as damage to infrastructure, regulations or change to markets. Although this paper presents a selection of model outputs, we have also pointed to some other research outputs for New Zealand that have been published in the last 15 years.

## Methods

### Climate projections

Climate projections for New Zealand were dynamically downscaled from the best-performing global general circulation models (GCMs) of the Coupled Model Intercomparison Project Phase 5 (CMIP-5) archive generated for the Intergovernmental Panel on Climate Change Fifth Assessment Report (IPCC-AR5, IPCC 2014). The six best-performing representative models for the New Zealand region were chosen for use in impact studies (Ministry for the Environment 2018). They are: HadGEM2-ES (UK), CESM1-CAM5 (USA), NorESM1-M (Norway), GFDL-CM3 (USA), GISSE2-R (USA) and BCC-CSM1.1 (China). Each model was bias-corrected and downscaled to the National Institute of Water and Atmospheric Research (NIWA) 5 km Virtual Climate Station Network grid (Sood 2014; Ministry for the Environment 2018). The downscaled data, including uncertainties, have been comprehensively analysed by Ministry for the Environment (2018). For computational reasons, the sheep facial eczema analysis described in this paper used one GCM (HadGEM2-ES), and the pasture production analysis used three GCMs. All the other analyses were based on the six GCMs. The horticultural analyses in this work used a different bias correction approach, as described by Vetharaniam et al. (2022b).

In line with IPCC-AR5, four scenarios of future greenhouse gas (GHG) emissions, or representative concentration pathways (RCPs), were selected. They are (in order of increasing atmospheric GHG concentrations) RCP 2.6, RCP 4.5, RCP 6.0 and RCP 8.5. RCP 2.6 is a low-end scenario consisting of aggressive emissions reductions and/or CO_2_ removal from the atmosphere. On the other end of the spectrum, RCP 8.5 is a high-end, worst-case scenario, with no mitigation of global GHG emissions, which would result in a global mean temperature increase of as much as +4°C by 2100 (IPCC 2014). RCP 4.5 and 6.0 are in between these two extremes. The RCP 8.5 scenario, though not very likely given its assumption of a lack of policy response to climate change, is a good illustration for a worst-case scenario that should be considered when planning for future change, given the high amount of uncertainty in carbon cycle feedbacks and socio-economic conditions (Kemp et al. 2022). We aim to provide results for all four RCPs. Projections of minimum and maximum temperature, precipitation, solar radiation, relative humidity, mean sea-level pressure and average wind speed are available at a daily time-step.

### Models

Different modelling approaches of contrasting complexity were selected to address identified risks, reflecting the available data and knowledge to assess specific climate change impacts (Table 2). We used six types of models with various degrees of complexity. The low complexity types (1 and 2) are quick to run in response to average climate inputs. Low-to-medium complexity models (types 3 and 4) involve expert knowledge and more spatially explicit data inputs. More complex models (types 5 and 6) involved mechanistic models (simplified dynamic to detailed, respectively) that can be challenging to run when simulating crop-soil-water processes on a daily time-step. Complex models are more likely to be able to represent the interactions between plants, soils and the environment. They can also inform more detailed quantitative outputs, such as yield, timing of phenological stages or water deficit, contingent on accurate specification of the model parameters. Most of the models were run on the high-performance computer (HPC) infrastructure for computational and/or climate data storage reasons.

**Table 2.** Types of models used in the climate change assessments.

Complexity	Type	Description	Risks/impact	Type of risk
Low	1. Simple climate attribute metrics	Uses knowledge of temperature thresholds for specific crops	Crop heat stress; grow degree days (GDD); frost risk	Chronic, acute
Low	2. Data-driven empirical model	Uses available data to derive an equation for current day, which future climate is then applied to	Sheep facial eczema; barber’s pole worm; dairy cattle heat stress	Chronic, acute
Low-medium	3. Rule-based model	Uses expert knowledge to derive suitability indices based on rules	Change in suitability, yields	Chronic
Low-medium	4. Conceptual/empirical model	Combines empirical models with expert knowledge	Water quality – sediment; change in wine phenological stages	Chronic
Medium	5. Simplified dynamic model	Combines knowledge to create simple mechanistic models where change in the timing of phenology stages due to temperature is also accounted for	Change in timing; drought	Acute
High	6. Mechanistic model	Uses a complex mechanistic model (APSIM^a^) with future climate data	Ryegrass/white clover pasture yield	Chronic

^a^ https://www.apsim.info/.

The models were applied to all of New Zealand using the climate projection data described above and soil information from S-map (Manaaki Whenua - Landcare Research 2020b) and the Fundamental Soil Layers (Manaaki Whenua - Landcare Research 2020a), as required. The 5 km grid underpinning the climate data means that the risk estimates are limited in spatial detail. They can be used at the district scale and above, but further information would be needed for use at the farm scale.

### Arable sector

Using the simple type 1 model, thresholds and sensitive periods were identified for up to three hazards (heat stress, frost risk, extreme rainfall) for six crops: maize, wheat, onions, peas, potatoes and chestnuts, based on defined date ranges in a simplified representation of the sensitive period (Table 3).

**Table 3.** Key climate hazards and sensitive periods for 6 arable crops.

Crop	Sensitive period (date)	Climate variables	Critical value	Hazard
Chestnuts	01 Sep–30 Nov	T_max_	>40°C	Heat stress
	15 Sep–31 Jan	T_min_	<−4°C	Frost
Onions	01 Jan–29 Feb	T_max_	>31°C	Heat stress
	01 Nov–30 Nov	Tmin	<0°C	Frost
	01 Jan–31 Mar	PCP	> 3 days in any 7 day with ≥ 5 mm/day	Extreme rainfall
Peas	01 Nov–29 Feb	T_max_	>30°C	Heat stress
	01 Sep–30 Nov	T_min_	<−2°C	Frost
Potatoes	01 Nov–29 Feb	T_max_	>20°C	Heat stress
	01 Nov–29 Feb	T_min_	<0°C	Frost
Wheat	15 Oct–15 Dec	T_max_	>25°C	Heat stress
Maize	15 Dec–15 Feb	T_max_	>30°	Heat stress

### Horticultural sector

Rule-based models (type 3) using a continuous or fuzzy logic approach (Vetharaniam et al. 2022a) that could be discretised were linked to the climate change projection information for the following crops: apple, avocado, blueberry, cherry, kiwifruit and two wine grape varieties.

In an earlier study on the impacts for viticulture, empirical models were combined with an expert assessment of the dominant process (type 4). For instance, expert knowledge about the influence of temperature on phenological stages was used to create an empirical model that helped project future flowering times for wine cultivars (Ausseil et al. 2021).

### Pastoral sector

Phillips et al. (2023) describe the empirical work for the type 2 model developed for predicting the risk of *Pseudopithomyces chartarum* sporulation in pastures, which can cause facial eczema in sheep. The historical occurrence of facial eczema was related to temperature and rainfall, then predictions were made.

A simple temperature humidity index (Davis et al. 2003) was generated, indicating increasing risk of heat stress for dairy cows for all RCPs. Another research project is developing a more specific model of the impact of heat stress on dairy production and economics.^2^

A more mechanistic representation of the response of plants or animals to increasing temperature (model type 5) enables the development of risk indices where changes in the timing of key phenological stages can be included. In particular, we were interested in the risk of drought in maize silage (and grain) that also allowed for changes in planting/harvest due to changes in temperature. For example, crops may mature sooner, leading to a change in the period when rainfall or irrigation water is critical. Sood et al. (in prep.) describe how they modified a simple Food and Agriculture Organization (FAO) water balance model to estimate water deficit (potential evapotranspiration deficit − PED). The FAO crop stages for maize were linked to thresholds of accumulated thermal time and a corresponding coefficient of water demand using expert knowledge. The climate time series was used to determine the phenological stage of a maize crop and thus its water demand, and the water balance model accumulated the water deficit over the period of growth and water demand.

APSIM (model type 6) was set up to simulate a ryegrass/white clover sward with and without fertiliser and irrigation (N fertiliser varied with plant demand and was capped at 200 kg N/ha/year and near-optimal irrigation). All RCPs have been run, but only for three GCMs (due to the computational demand). The output consisted of estimates of average potential yield over 20 year periods. The simulations used the standard AgPasture model set-up (with default parameters) describing a rotational defoliation (triggered by pasture biomass), with no management limitations and minimal inefficiencies. The simulations assumed no nutritional deficiencies other than N and neither the occurrence of any pest or disease. The model has been tested under New Zealand conditions and including climate variations (Li et al. 2014; Vogeler and Cichota 2016; Cichota et al. 2018).

Dominant erosion processes were modelled in a type 4 approach to predict the changes in sediment yields (Neverman et al. 2023).

## Results

Results from the modelling are presented below, by sector. All of the GIS layers mentioned below (and more) can be accessed from the Whitiwhiti Ora Data Supermarket at https://landuseopportunities.nz/.

### Arable sector

**Figure 2.**
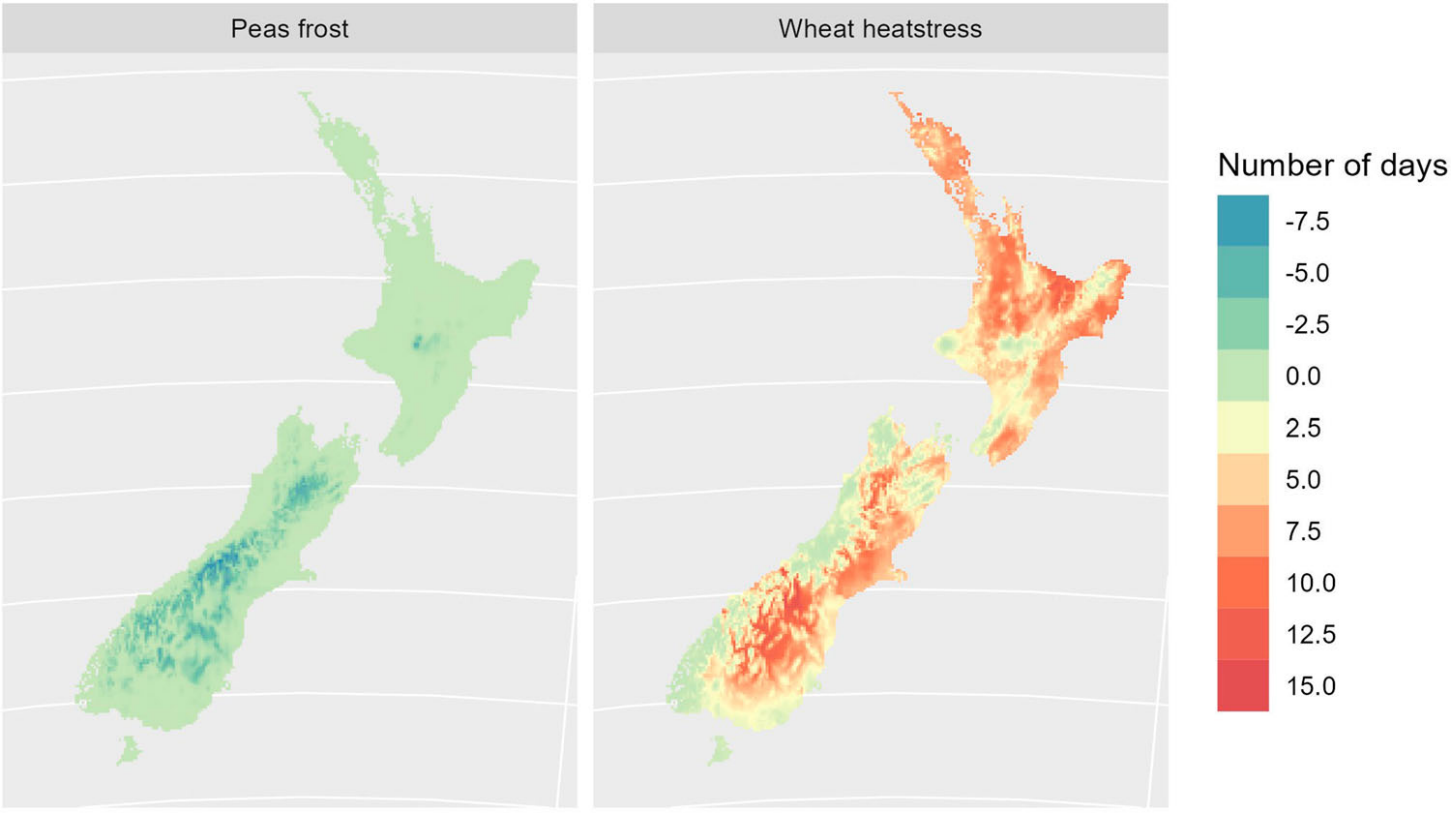
Change in risk of frost (peas) and heat stress (wheat) from a baseline (1985–2005) to a future climate (RCP 4.5, 2040–2060). Note that these risk maps must be used in conjunction with crop suitability maps that account for other climatic and soil requirements (see https://landuseopportunities.nz/).

Heat stress is becoming more of an issue (Figure 2). For example, on the Canterbury plains there is projected to be a mean increase of approximately six extra days per annum in which loss of yield could occur. Conversely, the lowering risk from frost damage means there will be new opportunities in some areas to plant crops such as onions, peas and potatoes.

### Horticultural sector

**Figure 3.**
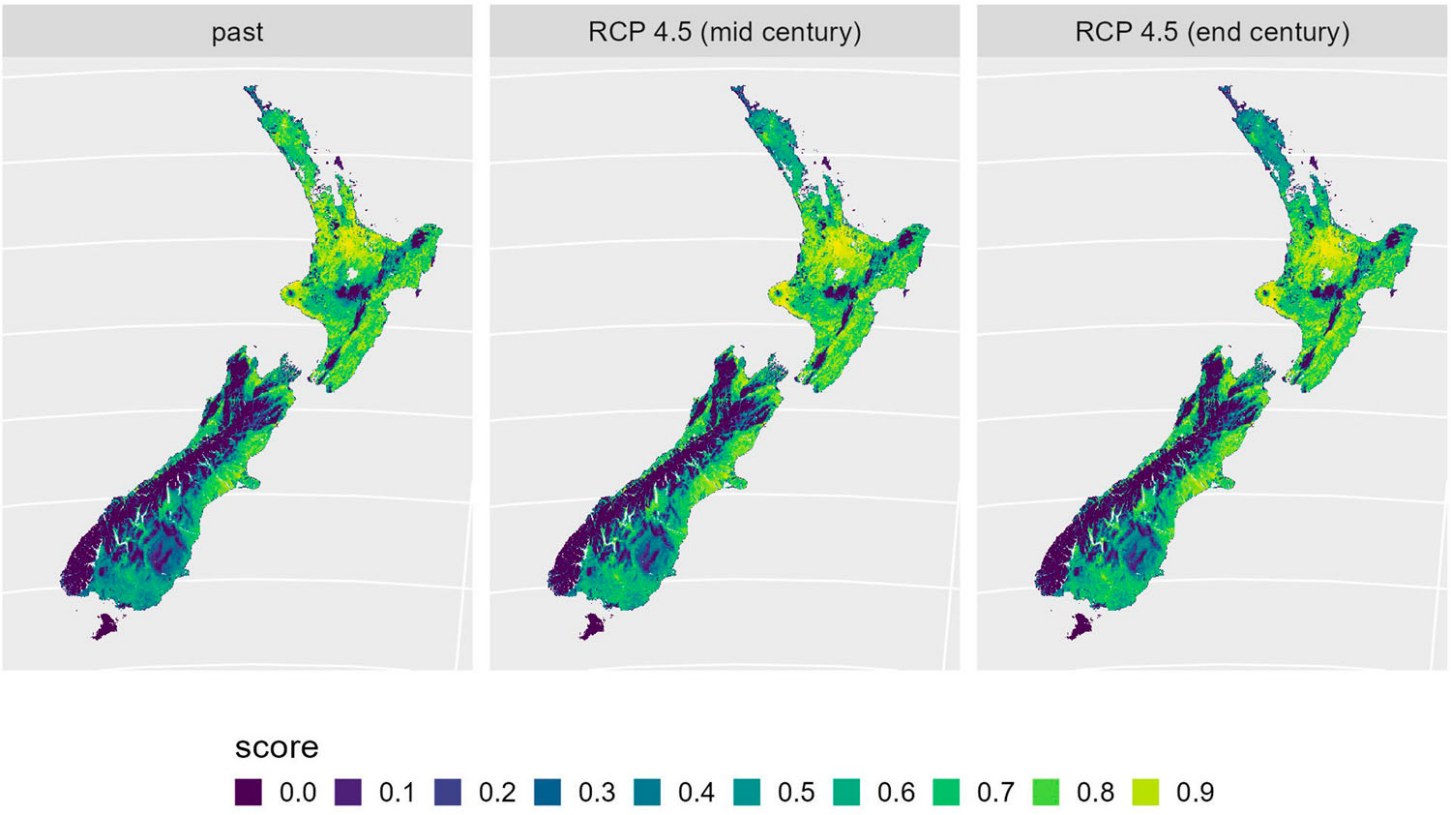
Suitability scores (0 = poor, 1 = good) for apples at the baseline period (1972−2004), mid-century (2028−2058) and end of century (2068−2098) under RCP 4.5.

The model results indicate a similar pattern of crop suitability moving south with time, while some previously suited land becomes less suited or unsuited. An example of apple suitability is shown in Figure 3.

The viticulture modelling showed that the phenological timing of bud burst and ripening was likely to advance, and that the timing between these stages varied among cultivars. This implies that different regional cultivars might ripen within a smaller window of time, complicating harvesting schedules across the country. It also suggested that New Zealand could consider either moving cool-climate cultivars further south (e.g. Sauvignon Blanc, Pinot Noir and Merlot), or using more late-ripening cultivars (although this would change the nature of the wine produced).

### Pastoral sector

**Figure 4.**
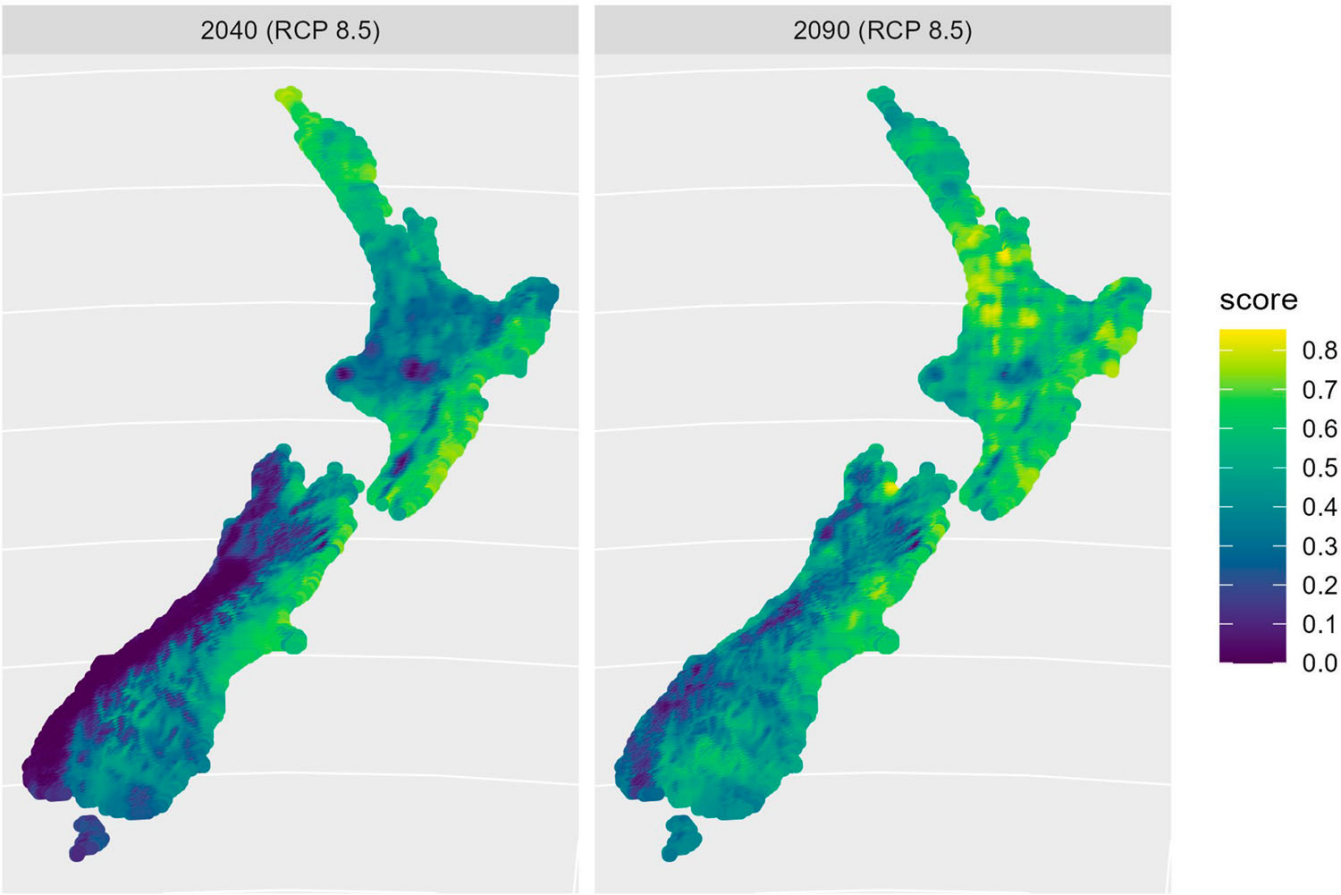
Predicted climate suitability for facial eczema in 2040 and 2080 under HADGEM2 emissions scenario RCP 8.5.

Even under RCP 2.6 the suitability for *P. chartarum* sporulation will increase with time in most regions, particularly in the North Island (Figure 4). The suitability for *Haemonchosis contortus*, a highly pathogenic intestinal nematode that affects sheep and cattle health, is similarly predicted to increase in the North Island and extend further south with time.

**Figure 5.**
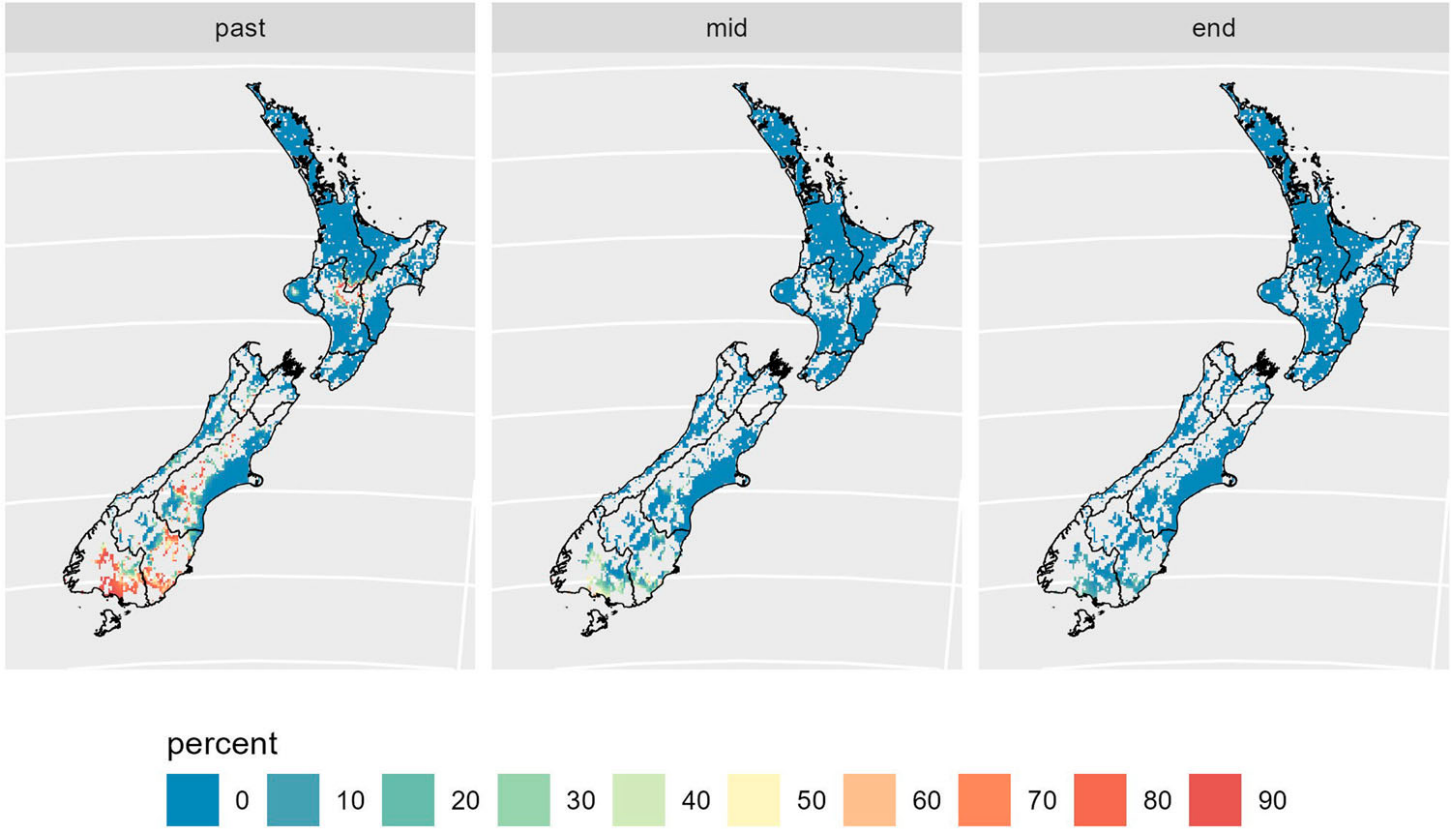
The percentage of 20 years where the maize silage crop did not reach maturity under baseline past (1981–2000), mid-(2041–2060) and late century (2080–2099) under RCP 4.5.

Figure 5 indicates that maize silage cropping may become more viable in southern regions as temperatures increase. Previous work with mechanistic models (model type 6) has shown similar patterns (Rutledge et al. 2017), with the crop also becoming more suitable at higher altitudes (Teixeira et al. 2018). This is a result of an increase in the growing season length as well as increases in daily temperature, with a reduction in the proportion of years with failed crops due to insufficient thermal units to complete the productive cycle, reflected in reduced inter-annual variability.

**Figure 6.**
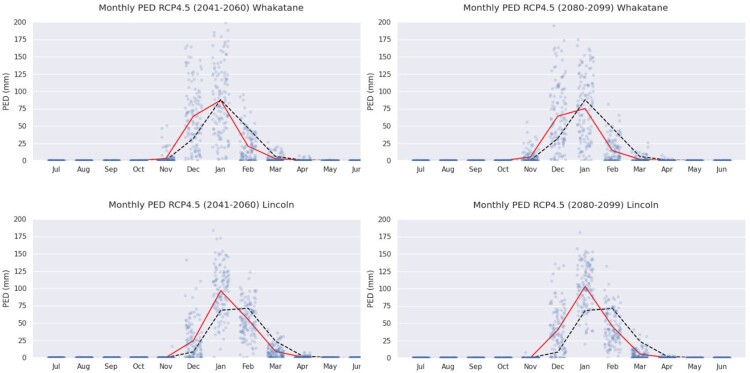
Monthly demand of maize crops for water at two locations (Whakatane RCP 4.5 and Lincoln RCP 4.5), and two time-slices (mid- and end of century). The dashed black line is the demand in the baseline past (1981–2000); the red line is the mean demand under RCP 4.5. The grey dots indicate the variability across the 20 year period and the six climate models.

Figure 6 shows how the peak period of water demand by maize crops occurs earlier in the season for more intense warming scenarios and late time-slices in the century (e.g. more demand in December for RCP 4.5 than in the past). Note though the variability in monthly drought predictions over the 20-year period (vertically) and the six GCMs (horizontally) as indicated by the grey dots (20×6).

Running our simple mechanistic FAO model with pasture shows increasing drought in the North Island in the summer, but spatially more variable impacts in the spring and autumn (maps not shown).

**Figure 7.**
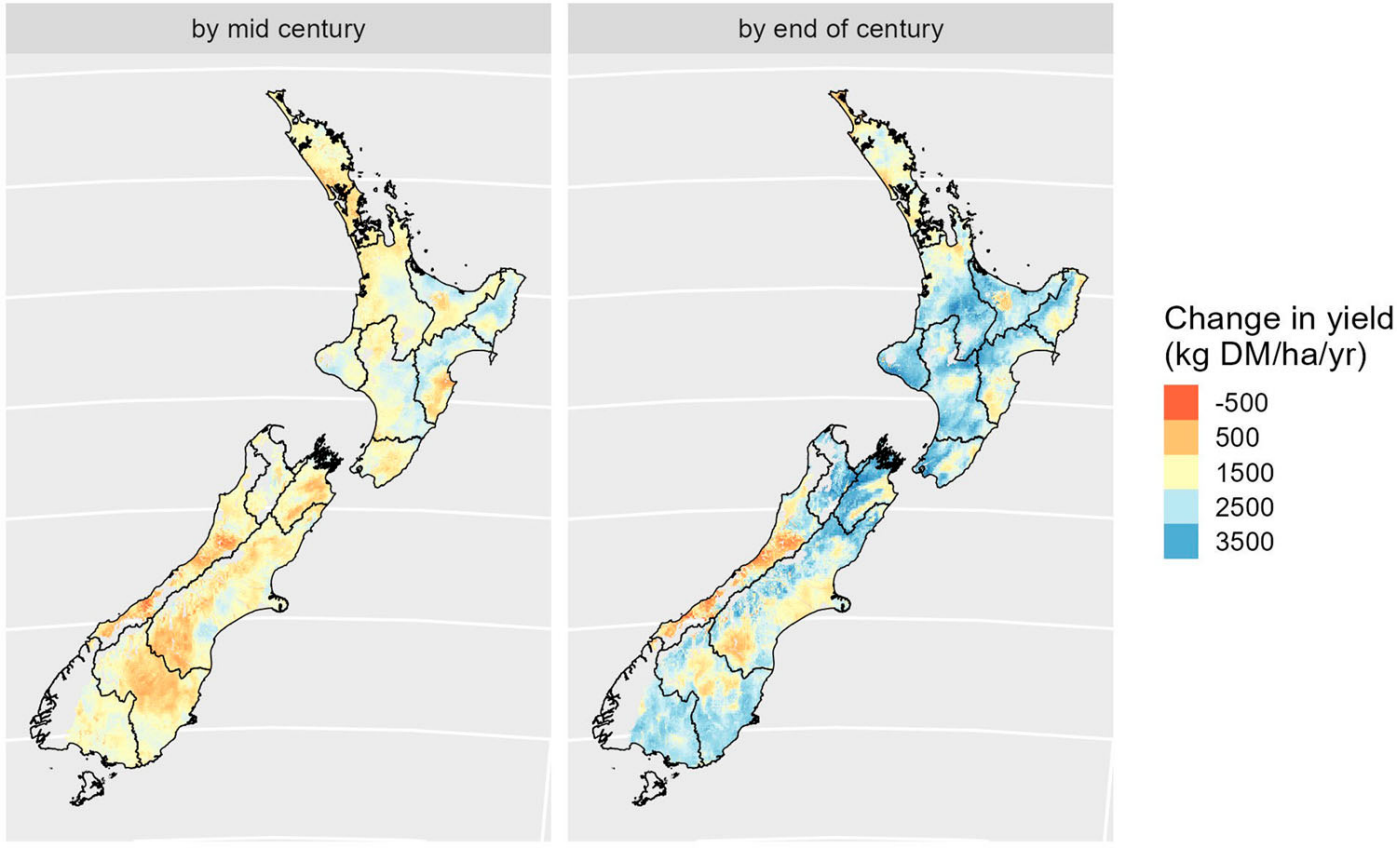
Projected changes in pasture yield at mid and end of century under RCP 6.0 (rainfed, no fertiliser).

Figure 7 maps the results of the Apsim simulations of pasture growth, showing that pasture yields may increase under RCP 6.0 in much of the country especially the central North Island and Southland by the end of the century, where limitations due to low temperatures become less pronounced. The simulations run for this work did not include changes in pasture species and did not consider the effect of slope and aspect, which can be important in hill country. The model seems to underestimate the effects of high temperatures in the upper North Island and may not have captured the full extent of water stress in the east.

**Figure 8.**
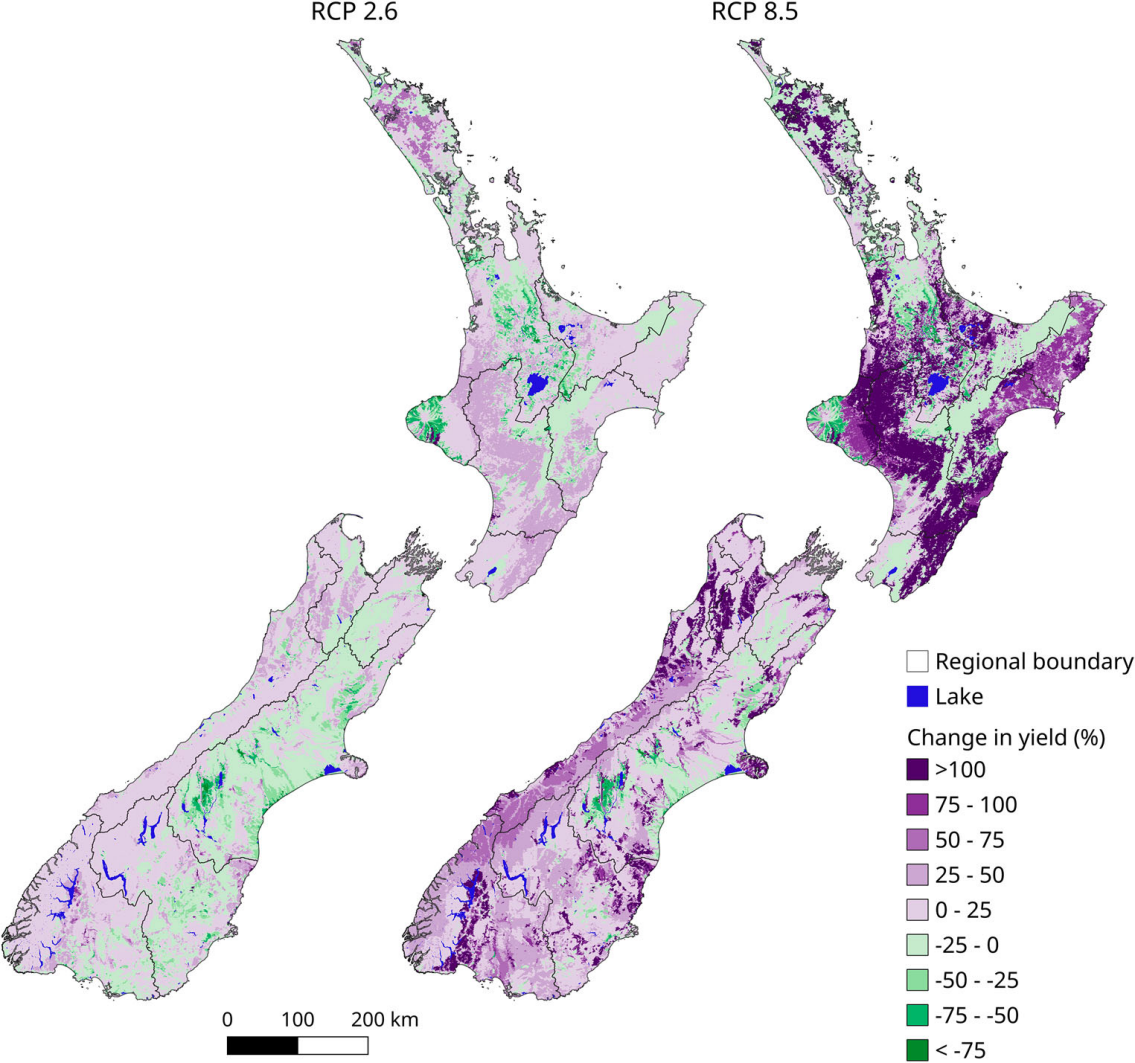
Median proportional change in total sediment yields across the six GCMs for the North Island and the South Island under RCP 2.6 and RCP 8.5 by 2090.

The study demonstrated a disproportionate increase in mass movement erosion expected in soft-rock hill country (Figure 8) with <1%–28% of North Island watersheds and <1%–8% of South Island watersheds estimated to experience a 100% increase in sediment yield by end of century, primarily driven by the impact of increasing storm magnitude frequency on mass movement erosion. This results in regional increases in sediment load delivered to the coast, ranging from 1% to 233%. Table 4 shows the variation in total load between regions and RCPs.

**Table 4.** Baseline total suspended sediment loads supplied to the coast of each region, and proportional change in total suspended sediment loads by mid-century under future climate scenarios.

Region	Baseline load (Mt year^−1^)	2040 Change in load (%)			
		RCP 2.6	RCP 4.5	RCP 6.0	RCP 8.5
Auckland	0.47	18	24	25	36
Bay of Plenty	6.2	16	21	21	29
Canterbury	9.4	6	7	11	10
Gisborne	28	23	31	30	40
Hawke’s Bay	10	24	29	30	41
Manawatu-Whanganui	12	33	45	44	59
Marlborough	1.2	7	6	7	4
Nelson	0.036	9	13	20	6
Northland	14	59	86	88	113
Otago	1.6	13	16	16	21
Southland	4.1	12	12	17	15
Taranaki	2.8	21	32	30	42
Tasman	2.4	13	15	21	21
Waikato	5.9	28	42	40	57
Wellington	5.2	36	47	47	61
West Coast	66	12	22	22	23

Source Neverman et al. (2023).

## Discussion

### Climate projections

A key advance described in this study is to use spatially resolved daily time series in the modelling. The high-resolution climate change maps of the climatological averages of key climate variables (e.g. temperature, precipitation) based on CMIP-5 future climate projections are available from the NIWA websites.^3^ These climatological averages are not suitable for generating more comprehensive information on the crop life-cycle changes, such as shifts in flowering and subsequent impacts on yields. Even though these maps represent extreme events (e.g. climatological changes in drought frequency and intensity, 99-percentile rainfall, and heatwaves), they are not informative enough to comprehensively determine event-driven risks such as heatwaves, cold spells or floods. Note, however, that even in the daily time series we have only a limited representation of the extreme events that are, by their nature, exceedingly rare in the short (20 year) time slices of non-stationary climate and a small ensemble of six GCM models. For example, the probability of a rare event (such as 1 in 100 year) occurring in a 20 year time slice is low, and a more reliable estimate will require a considerably larger model simulation ensemble.

Our models used all four RCPs from 2.6 to 8.5. While most research publications tend to present results for the worst-case scenarios, we intentionally chose to show results from a range of RCPs. Debate in the science community is still ongoing on the likelihood of following either the RCP 2.6 or the RCP 8.5 pathway (Sanderson et al. 2016; Hausfather and Peters 2020). Our results show that even under middle-of-the-range scenarios (RCP 4.5 and 6.0), impacts are likely to be significant and therefore need to be considered seriously.

Even though the next generation of CMIP-6-based climate projections is now becoming available, the main results derived in this study are likely to remain robust. It is essential that the key climate variables are regionally downscaled, evaluated and bias corrected with respect to observation-based data. The best available data in New Zealand is the Virtual Climate Station Network. This work is underway for CMIP-6 (Sood et al. 2020), and in our view shows that the climate change signal over model generations is likely to remain mostly qualitatively stable.

### Model types

Simple models can be sufficient to answer more general questions related to climate change. Expert knowledge can be elicited to support immediate decision-making by farmers and industry professionals. For instance, the simple crop suitability and hazard indices can help the farming industry to understand future land-use change opportunities across the country. However, for more specific questions, such as ‘What would be the projected changes in yield at a given location?’, more sophisticated models are required.

When model maturity is good enough to trust predictability, these models can inform potential variations in yield under given specific climate change scenarios. However, models vary in how they account for different processes, so it is important to ensure that outputs are accompanied by metadata on fitness for purpose and limitations (as described in the data supermarket: see metadata). The development of mechanistic models relies heavily on the level of understanding of specific biophysical processes, and then on implementing them within the model. Accurate parameterisation is another key challenge. This is labour-intensive, necessitating calibration with in-field measurements to ensure they have sufficient credibility in their predictive capacity to project into the future. Further research is still needed, for instance, to better understand the impacts of climate change on crop phenology, the dynamics of pests and diseases, or the magnitude of impact of increased CO_2_ on plant physiology (fertilisation effect, water demand, etc.).

Although simple models may not be suitable for some questions, more complex models also have drawbacks in terms of their computational power requirements, reliance on experts to run them, uncertainty of the parameterisation, and level of data inputs needed. Articulating the right questions and objectives, and assessing data availability and the appropriateness of existing models to evaluate climate change impacts are all necessary steps to enable informed decision-making (Vannier et al. 2022). Note that the model 5 type is a compromise approach that seeks to employ the advantages of a mechanistic model without the computational requirements and need for hard-to-obtain parameters.

### Modelling results

The various work streams have tried to address both chronic and acute risk questions. The modelled effects of climate change varied spatially across all crops and impact metrics. Some risks are significant for large areas; for example, the expected increase in sediment yield from the soft-rock hill country in the North Island could offset any land management improvements linked to water quality standards. Drought looks to be an increasing issue for rainfed pasture in the North Island. Drought risk for crops, such as maize, may be less significant than for pasture under climate change because crop sowing dates can be advanced under warmer climates, thus taking advantage of the spring rainfall and winter soil water storage. Other impacts are more localised to specific microclimates. For example, under climate change some locations will be less affected by the risk of frost, potentially reducing the probability of crop failure and offering new opportunities to grow some arable or horticultural crops.

### Future work

Although summary information like that presented here and available at the data supermarket (e.g. mean annual maps) can show the trends that can be expected as the climate changes, there is much to be gained from extending the analyses to inspect the inter-annual variability of risk as well as the temporal patterns of duration and frequency. For example, is the length of drought periods or their timing changing? What is the likelihood of multi-regional and consecutive-years droughts, and how are they going to affect the agricultural sector? Are the probabilities of adverse events changing? The information can also be used to investigate more farm-type specific questions (e.g. does an increase in pasture production mean that high-country farms will have more potential to finish their lambs?). Uncertainty could be more explicitly presented. A more temporally detailed analysis would allow separation and analysis of the various drivers, including the negative effects of drought in the summer vs the positive effects of CO_2_ fertilisation and warmer winters.

We suggest that the next steps should include an iterative process of working with farm systems scientists and agricultural experts to identify sets of questions about the future that relate to a range of different farm types, locations and commercial interests, which modellers can then seek to answer. Each iteration would enable the group to devise new questions, ensure the relevance of the answers, and help the agricultural sector to engage with the information.

Although there is uncertainty in both the climate projections themselves and in the accuracy of the modelled responses to climate change (Mackay et al. 2023), the information is still valuable for considering how to take advantage of the projected changes as well as identifying adaptative pathways for more resilient farms, especially those, such as land-use change, that will take time and investment to implement.

Adaptation to climate change is critical given the significance of the economic and social importance of the agricultural sector in New Zealand. Having quantitative information on potential risks and opportunities and implications can inform adaptation pathways for communities and avoid risks of maladaptation (Lawrence et al. 2024). Importantly, the physical impacts of climate change should be integrated and coupled with socio-economic models to assess wider implications to the community and sector. These may require more process-based models that can produce the inputs needed for economic models and respond to feedbacks from them (Ausseil et al. 2019b). Moreover, progress is still required to model the socio-economic implications of adaptation measures (Giupponi et al. 2022).

## Conclusions

This paper describes the latest research in understanding climate change impacts on New Zealand agriculture. A set of models ranging in complexity were developed and used with spatial data to advance our knowledge of risks and opportunities for arable, horticultural and pastoral land uses. This information is available on https://landuseopportunities.nz/. A more temporally detailed analysis of this information would allow more specific impact questions to be explored. Finally, more research in partnership with the agricultural sector is needed to help with adaptation planning and developing resilience to climate change. We hope these results will contribute to the next national CCRA due in 2026. Our results should inform discussions on land use and the balance with climatic risks in New Zealand. This will be helpful to policy makers, regulators, and land stewards, guiding decision-making on adaptation options (tactical, strategic or transformational) for long-term shifts in risks (e.g. change in cultivars) or the mitigation of risks (e.g. investment in water storage, breeding) (Ausseil et al. 2019b).

## Notes

1 https://www.tcfdhub.org/Downloads/pdfs/E06%20-%20Climate%20related%20risks%20and%20opportunities.pdf.

2 https://deepsouthchallenge.co.nz/resource/dairy-nz-matamata-piako/.

3 For example, https://ofcnz.niwa.co.nz/#/nationalMaps, https://niwa.co.nz/our-science/climate/information-and-resources/clivar/scenarios#ourfutureclimate.
